# Systematic Applications of Metabolomics in Metabolic Engineering

**DOI:** 10.3390/metabo2041090

**Published:** 2012-12-14

**Authors:** Robert A. Dromms, Mark P. Styczynski

**Affiliations:** 1 School of Chemical & Biomolecular Engineering, Georgia Institute of Technology, 311 Ferst Drive NW, Atlanta, GA 30332, USA; 2 Institute for Bioengineering & Bioscience, Georgia Institute of Technology, 315 Ferst Dr. NW, Atlanta, GA 30332, USA

**Keywords:** metabolomics, metabolic engineering, mass spectrometry, metabolic flux, metabolic profiling, principal components analysis, partial least squares regression, flux balance analysis, constraint-based models, kinetic ODE models

## Abstract

The goals of metabolic engineering are well-served by the biological information provided by metabolomics: information on how the cell is currently using its biochemical resources is perhaps one of the best ways to inform strategies to engineer a cell to produce a target compound. Using the analysis of extracellular or intracellular levels of the target compound (or a few closely related molecules) to drive metabolic engineering is quite common. However, there is surprisingly little systematic use of metabolomics datasets, which simultaneously measure hundreds of metabolites rather than just a few, for that same purpose. Here, we review the most common systematic approaches to integrating metabolite data with metabolic engineering, with emphasis on existing efforts to use whole-metabolome datasets. We then review some of the most common approaches for computational modeling of cell-wide metabolism, including constraint-based models, and discuss current computational approaches that explicitly use metabolomics data. We conclude with discussion of the broader potential of computational approaches that systematically use metabolomics data to drive metabolic engineering.

## Abbreviations

**Table:** 

Abbreviation	Meaning	Abbreviation	Meaning
CE-MS	Capillary Electrophoresis-Mass Spectrometry	MOMA	Minimization Of Metabolic Adjustment
CHO	Chinese Hamster Ovary	NET	Network-Embedded Thermodynamic
COBRA	Constraints Based Reconstruction and Analysis	NMR	Nuclear Magnetic Resonance
dFBA	Dynamic Flux Balance Analysis	OMNI	Optimal Metabolic Network Identification
EMUs	Elementary Metabolite Units	ODE	Ordinary Differential Equation
FBA	Flux Balance Analysis	PLS	Partial Least Squares
GC-MS	Gas Chromatography-Mass Spectrometry	PLS-DA	Partial Least Squares Discriminant Analysis
HCA	Hierarchical Clustering Analysis	PCA	Principal Components Analysis
HPLC	High-Performance Liquid Chromatography	QP	Quadratic Programming
idFBA	Integrated-Dynamic Flux Balance Analysis	rFBA	Regulatory Flux Balance Analysis
iFBA	Integrated Flux Balance Analysis	SBRT	Systems Biology Research Tool
IOMA	Integrative “Omics”-Metabolic Analysis	TMFA	Thermodynamic Metabolic Flux Analysis
LP	Linear Programming	TAL	Transaldolase
LC-MS	Liquid Chromatography-Mass Spectrometry	TKL	Transketolase
MASS	Mass Action Stoichiometric Simulation	TCA	Tricarboxylic Acid
MCA	Metabolic Control Analysis	VIP	Variable Importance in the Projection
MFA	Metabolic Flux Analysis	VHG	Very High Gravity

## 1. Introduction

Organisms such as *Saccharomyces cerevisiae* and *Aspergillus niger* have a long history of commercial use in natural fermentation processes to produce chemicals such as ethanol and citric acid. Traditional bioprocess engineering entails the design and optimization of the equipment and procedures necessary to efficiently manufacture these and other biologically derived products. The development of recombinant DNA technologies enabled the direct manipulation and expansion of the metabolic capabilities of *S. cerevisiae* and *A. niger* (as well as other organisms such as *Escherichia coli* and *Bacillus subtilis*), which resulted in the emergence of metabolic engineering as a field distinct from bioprocess engineering [1]. Metabolic engineering is the (usually genetic) control of the metabolic activities of a living organism to establish and optimize the production of desirable metabolites – the class of small molecules that comprise the primary resources and intermediates of all cellular activity. With widespread and growing interest in environmentally sustainable industrial technologies, metabolic engineering is poised to provide an effective and efficient means for producing various small molecule chemicals from clean and renewable sources, such as biofuels derived from lignocellulosic feedstock [2,3,4,5,6,7,8,9,10,11,12].

Frequently, metabolic engineering studies use targeted analysis of a few carefully selected intracellular or secreted extracellular compounds to drive or assess the progress of their efforts [2,9,13,14,15,16,17,18,19,20,21,22,23,24]. High-Performance Liquid Chromatography (HPLC) and enzymatic assays have typically been the methods of choice to generate this data, used in engineering *S. cerevisiae* [2,13,21,23], *E. coli* [16,19], *Clostridium acetobutylicum* [14,18], and other organisms. These measurements may be direct readouts of the performance of an engineered strain [2], or they may be interpreted as performance and response characteristics (for example, trehalose as a marker for stress response in yeast [21,23]). These analyses are typically focused on effects at the level of individual pathways [2,19,21,25].

Another technique used to characterize metabolic pathways during metabolic engineering is Metabolic Flux Analysis (MFA). MFA provides more information than measurement of just a few metabolites, and is a staple technique of many who work in metabolic engineering [14,18,20,22,24,26,27,28,29,30,31,32]. In MFA, isotopically labeled metabolites (typically using ^13^C labels) are leveraged to calculate fluxes – the rate at which material is processed through a metabolic pathway – from knowledge of carbon-carbon transitions in each reaction and the measured isotopomer distribution in each metabolite [1]. Ongoing research in MFA includes continued improvement of ^13^C protocols and analytical platforms [33,34,35,36], improvements to software for MFA calculations [32,37], use of network stoichiometry to determine the minimal set of required metabolite measurements [38], and study of Elementary Metabolite Units (EMUs) for more efficient analysis of flux patterns [31,39,40].

Metabolic engineering seeks to maximize the production of selected metabolites in a cell, whether produced by the organisms’ natural metabolic activities or by entire exogenous pathways introduced through genetic engineering. Strategic, small-scale measurements and flux calculations have to date been indispensable tools for metabolic engineering. However, the development of systems-level analyses – precipitated by whole-genome sequencing and the rapid accumulation of data on RNA, protein and metabolite levels – has provided new opportunities to more completely understand the effects of strain manipulations. Genetic modifications often have additional effects outside the immediately targeted pathway, and a better understanding of the nature and extent of these perturbations would lead to more effective strategies for redesigning strains, as well as improved ability to understand why a proposed design may fail to achieve its predicted performance.

Aided by recent advancements in analytical platforms that allow for the simultaneous measurement of a wide spectrum of metabolites, metabolomics (the analysis of the total metabolic content of living systems) is approaching the level of maturity of preceding “global analysis” fields like proteomics and transcriptomics [41,42]. Metabolomics approaches have already found some success in clinical applications, where studies have demonstrated their efficacy in identifying clinically relevant biomarkers in diseases such as cancer [43,44,45]. Surprisingly, though, the application of metabolomics approaches to problems in metabolic engineering has been somewhat scarce.

Here, we review examples of recent strategies to integrate metabolomics datasets into metabolic engineering. First, we briefly cover the fundamentals of metabolomics. We then discuss strategies for assessing metabolic engineering strain designs, and how metabolomics methods can extend these strategies. We follow with discussion of computational tools for metabolic engineering, with an emphasis on how these methods are used to design strains and predict their performance as well as how metabolomics datasets are currently applied to computational modeling. We conclude with a brief summary of the state of the field and the potential that integrating metabolomics presents.

## 2. Metabolomics Background

The development of metabolomics, the newest of the global analysis methods, has much in common with its predecessor fields of genomics, transcriptomics, and proteomics [41,42]. The analytical platforms used for metabolomics have now developed to the point that metabolomics datasets can serve as an excellent complement to standard metabolic engineering approaches. The goals of metabolic engineering ultimately focus on producing desired metabolites, and metabolomics offers a means of broadly and directly assessing how well a strain meets those goals. What follows is a cursory overview of metabolomics technologies and the most common ways that metabolomics data are interpreted and analyzed, provided as context for how metabolomics data can be used towards metabolic engineering efforts.

### 2.1. Analytical Platforms

One of the primary difficulties facing the development of metabolomics has been the staggering diversity of metabolites. Metabolites are substantially more chemically diverse than the subunit-based chemistries of DNA, RNA, and proteins, impeding the progress of metabolomics as a truly “omics” field that measures all metabolites. The entire genome and transcriptome can be (at least theoretically) surveyed using single platforms, from simple PCR to more exhaustive sequencing and microarrays, whereas metabolomics requires multiple analytical platforms to achieve complete coverage of all metabolites.

Common approaches involve coupling of a chromatographic separation to mass spectrometry, including gas chromatography-mass spectrometry (GC-MS) [6,25,28,29,34,46,47,48,49,50,51,52,53,54,55,56,57,58], liquid chromatography-mass spectrometry (LC-MS) [26,33,34,49,52,54,56,59,60,61,62,63], and capillary electrophoresis-mass spectrometry (CE-MS) [34,63,64,65]. Other common platforms include nuclear magnetic resonance (NMR) [28,43,66,67] and an assortment of direct injection-mass spectrometry methods [43,48,51,53,67]. Protocols for using these platforms are under constant development, and span sample processing and work-up [51,56,68], efforts to improve the quantitative reliability of measurements [33,56,61], and data processing software [69,70,71,72,73,74,75,76,77,78]. These software tools, along with those presented in subsequent sections of this manuscript, are summarized in Table 1 (though we emphasize that this list is far from exhaustive). A more extensive review of these platforms is available from Dunn *et al.* [79]. 

### 2.2. Data Analysis

**Figure 1:**
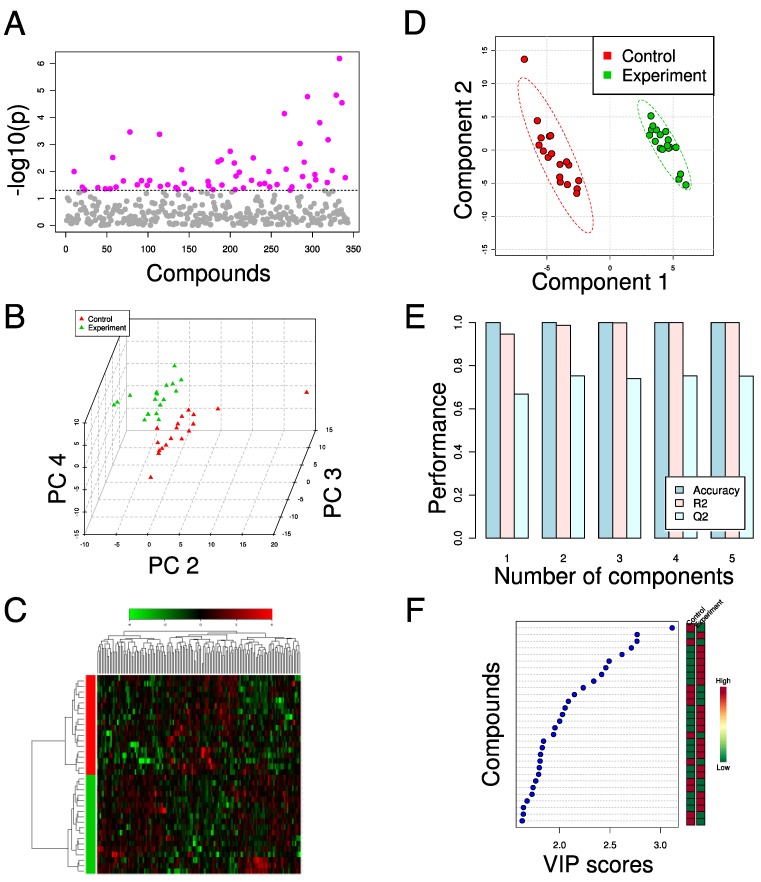
Examples of data analysis techniques for metabolomics. The effects of glucose deprivation on a cancer cell line were measured with GC-MS and analyzed in MetaboAnalyst [78] (unpublished data). (**a**) Pairwise t-tests of metabolites identify statistical significance of differences in individual compounds between control and experiment. The dotted line indicates p < 0.05 (no multiple hypotheses testing corrections). (**b**) PCA score plot reveals separation between control and experiment samples in components 2, 3, and 4. Component 1 (not shown) corresponds to analytical batch separation. (**c**) HCA (Ward method, Pearson’s correlation) and heatmap using the 150 most significant compounds as determined by t-test. Compounds along top, samples along left side. (**d**) PLS-DA score plot shows separation achieved using components 1 and 2. Dashed circles indicate the 95% confidence interval for each class. (**e**) Leave-one-out cross-validation shows that the majority of the predictive capacity is derived from the first two PLS-DA components. R^2^ and Q^2^ denote, respectively, the goodness of fit and goodness of prediction statistics. (**f**) Contribution of individual compounds to PLS-DA component 1. The 30 most important compounds and their relative abundance in control and experiment are shown, sorted by the Variable Importance in the Projection (VIP) [91] for the first component.

As the youngest of the global analysis methods, metabolomics has drawn heavily from the data analysis techniques developed for transcriptomics and proteomics. Like these two fields, the datasets generated by metabolomics suffer from a “curse of dimensionality,” where there are far more variables than there are samples. Methods taken from transcriptomics and proteomics, as well as some derived from the field of chemometrics, have been used extensively to analyze metabolomics data as a result [41,42] (some examples given in Figure 1). Though metabolomics datasets are of high dimension, the connected nature of biochemical pathways and networks can often lead to strong underlying patterns in the data; multivariate techniques have proven effective at identifying these underlying factors even if individual effect sizes are too small to be detected by univariate analyses (e.g., Figure 1a), and so here we highlight several of the methods most widely adopted for metabolomics studies.

One of the most prominent methods for analysis of metabolomics data is Principal Components Analysis (PCA) (Figure 1b). This technique identifies the natural “axes” of variation in the dataset by constructing a series of orthogonal component axes from the original metabolite features. Each component is a weighted combination of the original metabolite measurements that provides the maximum possible variance in a single composite variable; the components are all mutually orthogonal. The weights of the original features for each component (“loadings”) and the projections of the samples onto the components (“scores”) can reveal putative biomarkers or lead to simplified separation between biological sample classes, respectively [41,48,49,57,68,80,81,82,83,84,85,86]. Notably, this is an unsupervised technique; PCA uses no information about sample classes in its calculations, and the user can try to identify clusters of data points before projecting class information onto the score plot.

A few examples of using PCA to reveal underlying patterns in metabolomics datasets include the characterization of extracellular culture conditions in Chinese Hamster Ovary (CHO) cell batch cultures [85], a study of the response of *S. cerevisiae* to very high gravity (VHG) fermentations [80], comparisons of metabolomes across mutant strains of *S. cerevisiae* [42,48,68,82], and analyses of *Pseudomonas putida* growth on various carbon sources [49,81]. In this context, the loadings from the components that capture the separation between sample classes (e.g., culture condition or strain) on the score plot provide information about which metabolites are important to each class. The magnitude of each metabolite’s loading coefficient, and the groups of metabolites with high loadings in components that capture separation, can be used to infer biological significance.

Much of the value of PCA comes from its dimensional reduction capabilities: typically the first few components contain biologically relevant information, and higher components contain variance due to noise or biological variability. The number of components that are “significant” is an open question, and depends predominantly on the dataset or even the specific downstream processing and applications [87]. Since the principal component scores are “optimal” lower-dimension projections of the original data, they can be used in place of the original data in subsequent analysis, such as Hierarchical Clustering Analysis (HCA, Figure 1c) [85]. For example, Barrett *et al.* performed PCA on a flux balance analysis solution space to identify a lower-dimension set of key reactions that form the underlying basis of the solution space [84].

Partial Least Squares (PLS) regression and discriminant analysis (PLS-DA, Figure 1d-f) are also common tools in metabolomics analysis. They are multivariate analogs of linear regression and linear discriminant analysis, respectively. They are constructed in a manner similar to PCA, but require response variables (e.g. titer, viability, or conversion) or a class label, respectively, to determine the component axes [88]. Again, assessment of metabolite loading coefficients in PLS-DA axes allows biological interpretability. In one representative application, Cajka *et al.* used PLS-DA to identify a set of compounds that could discriminate between different beers by their origin [67]. Kamei *et al.* used OPLS-DA, a variant of PLS-DA that constructs distinct predictive and orthogonal components that describe between-class and within-class variance, respectively, [89,90], to assess the effects of knockouts related to replicative lifespan in *S. cerevisiae* [86]. They found that a component corresponding to separation between short-lived and long-lived strains identified differences in TCA cycle metabolites as predictors of longevity. These are just a few examples of the increasingly prevalent applications of PLS-based techniques in the field.

Complete and effective use of a metabolomics dataset necessitates not only careful design of experiment and data processing methods, but also a thorough validation of conclusions from data analysis (e.g. apparent clusters in principal component space). For example, discussion of p-value distributions by Hojer-Pedersen *et al.* touches upon the importance of multiple hypothesis testing corrections in metabolomics studies, such as Bonferroni or false discovery rate corrections [82,92]. As a supervised method, PLS-DA is particularly susceptible to over-fitting, and so cross-validation is critical [93]. Statistical issues aside, non-biological factors can also lead to separation in principal component space, with sources of variance potentially including derivatization protocols [68,80,81], analytical platform [48], chromatographic drift or batch effects [94] and data processing methods [81]. Broadhurst and Kell review other potential pitfalls in greater detail [95].

## 3. Applications of Metabolomics in Metabolic Engineering

Metabolomics continues to be exploited for numerous biomedical applications, ranging from the study of differences between clinically isolated and industrial yeast strains [83], to blood or urine-based biomarkers for many human diseases, including diabetes [64,96], gallstone diseases [97], and multiple types of cancer [43,44,45] (Blekherman *et al.* provide a more comprehensive review of the applications of metabolomics to cancer biomarker discovery [98]). Metabolomics also has the potential for a significant biotechnological impact in metabolic engineering: as the goal of metabolic engineering is to manipulate metabolite production, metabolomics naturally lends itself to that goal. Moreover, organisms such as *S. cerevisiae* and *E. coli* have been studied extensively, providing a rich biological context in which the metabolome of strains derived from both rational design and directed evolution strategies can be interpreted and understood. Nonetheless, the use of metabolomics in metabolic engineering is not as prevalent as one might expect.

### 3.1. Metabolomics Data as an Extension of Small-Scale, Targeted Analysis

The simplest and most direct use of metabolomics datasets is as an extension of existing small-scale metabolite analyses; metabolomics inherently enables a more comprehensive assessment of a strain than a handful of narrowly selected measurements. Studies employing this approach typically either compare strains and culture conditions, or seek to monitor the time-course evolution of many metabolite concentrations in parallel. These studies use a combination of measured growth and production parameters in conjunction with direct examination of the metabolomics data (e.g. significant increases or decreases in metabolite levels) in the context of known biochemical pathways to determine the effects of mutations and culture conditions. For example, if one overexpresses the enzyme that is the first step in a linear biosynthetic pathway and finds that the first few metabolites accumulate significantly but subsequent metabolites do not, this may suggest a rate-limiting step further down the pathway that needs to be upregulated. Broader knowledge of metabolite levels beyond the target pathway can serve to determine the wider-ranging effects of a given metabolic engineering perturbation and can suggest candidate supplementary perturbations (to address, for example, cofactor imbalances). 

One example of a strain- or condition-comparison approach is a study of an arcA mutant in *E. coli* by Toya *et al.*, which compared parent and mutant responses to aerobic, anaerobic and nitrate-rich media conditions [65]. Through analysis of fold-changes in the metabolome, transcriptome, and ^13^C MFA-derived fluxes, they found significant differences in tricarboxylic acid (TCA) cycle metabolism and ATP production among conditions. Similarly, Christen *et al.* compared the metabolomic profiles of seven yeast species to assess differences in aerobic fermentation on glucose [62]. While ^13^C MFA suggested differences between TCA cycle fluxes and consistent flux through glycolysis, there was a much wider variation of metabolite levels across species – especially in amino acid pool compositions. They also found that across species, these values correlated poorly with fluxes.

In an example of time-course analysis, Hasunuma *et al.* studied the effects of acetic and formic acid, chemicals commonly found in lignocellulosic hydrolysates, on xylose-utilizing strains of *S. cerevisiae* [10]. They engineered strains to overexpress transaldolase (TAL) or transketolase (TKL), which are thought to control rate-limiting steps in the pentose phosphate pathway during xylose utilization. Differences in intracellular levels of pentose phosphate pathway metabolites between the controls and the mutants as determined from fold changes confirmed the pentose phosphate pathway as a key chokepoint. Compared to the parent strain, they found that the single mutants both exhibited improved growth characteristics and ethanol production, while the double mutant did not. A separate similar study also explored differences in xylose-utilizing strains [9].

Other work with *S. cerevisiae* has investigated the transient effects of redox perturbations [99] or relief from glucose limitation [26,100], as well as differences between *S. cerevisiae* and *Pichia pastoris* [56]. However, examples span a variety of organisms and culture conditions, from xylose utilization in *A. niger* [6] to the effects of extended culture periods [101] and low phenylacetic acid conditions after key pathway knockouts [29] on penicillin biosynthesis in *Penicillium chrysogenum*.

### 3.2. General Strategies for Integrating Metabolomics into Metabolic Engineering

The simple approaches used to exploit the results of targeted measurements can be scaled up to metabolomics datasets, but they often do not take full advantage of structures or patterns in the data at the systems level. Many in the field of metabolic engineering have used multivariate techniques to interrogate metabolomics datasets on more complicated questions about strain performance and metabolite allocation. Due to the complexity of biological systems, the answers to these questions are often non-intuitive and increasingly difficult to identify without taking such a systems-scale approach. 

#### 3.2.1. Adaptive Evolution and High Throughput Libraries: Locating the Cause of Improved Phenotypes

While rational design approaches were the original driving force in metabolic engineering, directed evolution and high throughput screens of mutant libraries have since become increasingly commonplace [3,4,8,11,12,102,103,104,105,106,107,108,109]. One of the main difficulties involved in these two “inverse metabolic engineering” approaches is the identification of the mutations responsible for the improved phenotype [110]. These frequently non-intuitive changes can often best be pinpointed with a direct, systems-scale readout of the metabolic state [107].

Common techniques employed in such approaches include HCA, PCA, and PLS-DA. These methods generate clusters or loadings that identify key metabolite differences, which in turn suggest what genetic changes may have been selected for. For example, Hong *et al.* used PCA and clustering analysis of metabolomics data, supplemented with transcriptional data, to investigate strains of *S. cerevisiae* that had been selected via directed evolution for improved galactose uptake [107]. They identified up-regulation of PGM2 as a common method for indirectly improving flux through the Leloir pathway for galactose utilization via relieved feed-forward inhibition of galactose-1-phospate and activation of reserve carbohydrate metabolism. The enrichment analysis methods used for the transcriptional data are discussed in further detail in the next section.

A study by Yoshida *et al.* examined metabolic differences between *S. cerevisiae* and *Saccharomyces pastorianus* in regards to SO_2_ and H_2_S production [111]. They generated metabolomic and transcriptomic datasets under typical beer fermentation conditions, and identified lowered transcription levels and increased levels of upstream metabolites for two reactions in *S. cerevisiae* that they subsequently hypothesized to be rate-limiting fluxes for desired production of H_2_S and SO_2_. They verified their prediction by exposing *S. pastorianus* to known inhibitors for the corresponding enzymes, which recapitulated this effect. They used this knowledge to engineer and test *S. cerevisiae* strains that relieved these bottlenecks, and then developed a mutant with similar properties via a directed evolution process for commercial use.

Similarly, Wisselink *et al.* investigated a xylose-utilizing strain of *S. cerevisiae* developed by introduction of L-arabinose pathway genes to an existing xylose-utilizing strain, followed by directed evolution to improve L-arabinose utilization [112]. They used fold-change and enrichment analysis of transcripts, coupled with thermodynamic analysis of reactions using metabolite mass action ratios and reaction driving forces, to identify an acquired response of the GAL regulon to arabinose levels as contributing to these improvements. They confirmed this discovery with subsequent knockouts of GAL2.

Other examples of using metabolomics for after-the-fact assessment of engineered strains include studying the effects of repeated exposure to vacuum fermentation conditions on *S. cerevisiae* [113], comparing evolved strains with knockouts proposed by the OptKnock algorithm [47], and identifying the differences between several yeast species during aerobic fermentation on glucose [62]. 

#### 3.2.2. Other Global Analysis Approaches: Harnessing Proteomics, Transcriptomics, and Genomics for Metabolic Engineering

While the goal of metabolic engineering is to introduce a change on the metabolic level, many of these changes are necessarily implemented by introducing genetic modifications to affect transcriptional levels. As such, analysis of biological layers beyond the metabolome, such as the transcriptome and proteome, can provide further, and sometimes crucial, insight into the wide-reaching effects of an alteration.

A number of techniques widely used in metabolomics (such as PCA and HCA) are also well-established for many of these other “omics” datasets, though there are a number of other techniques that until recently were more specific to transcriptional or proteomic analyses. One of the most prominent examples of this is enrichment analysis, originally developed for transcriptomics datasets. Enrichment analysis uses information about the frequency of occurrence or the ranking of sets of gene names or functions in a given list of genes to examine the biological relevance of observed changes [114]. For example, the number of genes from a given pathway occurring in a list of interest (say, high-importance variables in PCA or a cluster from HCA) is assessed to see if that number of genes would be expected to be found in an arbitrary list of genes purely at random; this comparison is made using a hypergeometric distribution. If a list is statistically significantly “enriched” for a set of genes, one then may hypothesize that the list of genes plays an important role in the underlying biological process. This technique has recently been extended to metabolomics datasets [78,115]. A combination of enrichment, multivariate, and univariate analyses comprise the bulk of the strategies currently used in metabolic engineering to analyze “omics” datasets in parallel.

In metabolic engineering, use of metabolomics is comparatively much less common than using other global analysis approaches, perhaps attributable to the maturity of fields like transcriptomics and proteomics compared to metabolomics. Proteomics, transcriptomics and genomics have frequently been combined with small-scale metabolite measurements for metabolic engineering purposes. Examples of this include functional genomics with targeted metabolite measurements for isoprenoid production in *E. coli* [116], as well as the combination of the proteome, transcriptome and targeted metabolite measurements for *E. coli* carbon storage regulation [63], penicillin production in *P. chrysogenum* [101], glucose repression in *S. cerevisiae* [52], and relief from glucose deprivation in *S. cerevisiae* [100].

Flux measurements have also been commonly combined with other “omics” datasets, such as with the transcriptome and proteome in an analysis of lysine-producing *Corynebacterium glutamicum* during different stages of batch culture [46]. Other examples include the use of transcript data and ^13^C MFA to understand metabolic behavior in *A. niger* [7], transcriptional changes [117] and ^13^C MFA-derived fluxes [18] of *C. acetobutylicum* in response to stresses in bioreactors, and analysis of both systems-level MFA calculations [30] and Elementary Flux Modes [118] in tandem with transcriptomics data to assess metabolic control in *S. cerevisiae*.

The above examples at most used small-scale metabolite measurements, but a handful of studies have combined analysis of full metabolomics datasets with other “omics” datasets. Previously described analysis of adaptations from directed evolution generally fit this category: transcriptional measurements using microarrays [47,107,111,112] and genomic analysis [83] have each been combined with metabolomics to pinpoint the source of the observed phenotype.

In other applications, Piddock *et al.* assessed high gravity beer brewing conditions to determine the effect of the protease enzyme Flavourzyme on the free amino nitrogen content of the wort, and the resulting metabolomic and transcriptomic differences in a strain of brewer’s yeast [55]. A collaborative study by Canelas *et al.* investigated the growth characteristics of two strains of *S. cerevisiae* under two standard growth conditions. [66]. Datasets included intra- and extra-cellular metabolomes, the transcriptome, and the proteome, with an emphasis on reliable and quantitative measurements. They identified the specific growth rate and biomass yield differences between the strains that they attributed to differences in protein metabolism using analysis of variance (which determines statistical significance from comparisons of within-class and between-class variances) and gene enrichment analysis. Work by Dikicioglu *et al.* examined the combined metabolomic and transcriptomic response of *S. cerevisiae* to transient perturbations in glucose and ammonium concentrations [58]. Their work used HCA, t-tests, and analysis of fold-changes to study the short-term reorganization of the metabolome and transcriptome in response to temporary relief of nutrient-deprived conditions.

The emergence of genome-scale investigations has led to a deluge of information about all molecular layers in the cell. This in turn has provided a broader context in which metabolic engineering strategies can be evaluated. However, we note that many of the techniques discussed so far have focused on systematic *assessment* of the results of metabolic engineering strategies, rather than on systematic methods of *designing* strains to begin with. While some of these studies have used the insight gained from their evaluations to in turn design new strains, more systematic approaches to strain design are being developed – some of which are now capable of exploiting metabolomics datasets.

## 4. Computational Methods for Combining Metabolomics and Metabolic Engineering

**Figure 2:**
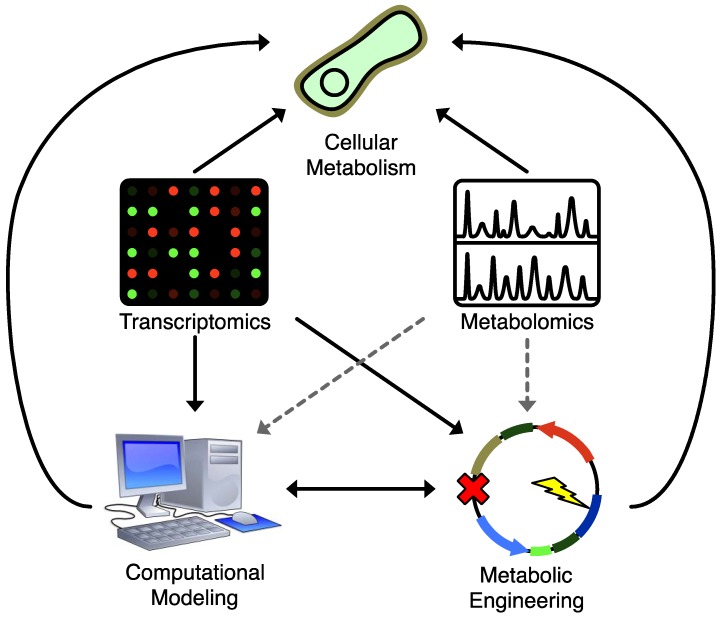
Applications of various techniques to understanding and manipulating cellular metabolism. Solid lines represent widely used strategies, dashed lines represent underused strategies. Both metabolomics and transcriptional profiling provide a direct readout that helps enable a deeper understanding of cellular metabolism, but only transcriptional profiling has seen widespread application to enhance standard computational modeling and metabolic engineering strategies. Integrating metabolomics data into metabolic engineering and computational modeling strategies would help bridge gaps in biochemical knowledge and improve our ability to control cellular metabolism.

One of the difficulties in applying metabolomics datasets to strain design is the volume of data produced in a metabolomics experiment. Computational approaches are well suited to systematically integrate large volumes of biochemical knowledge and data. As shown in Figure 2, they serve dual purposes: they can combine existing biochemical knowledge with strain design objectives to execute putative metabolic engineering strategies *in silico* before taking the time and expense to execute them *in vivo*, and they can close the loop on experimental design by producing hypotheses that, when tested, can be used iteratively to refine broader biochemical knowledge and models. This in turn leads to improved predictive power for subsequent rounds of metabolic engineering design.

One of the most powerful ways to extend this concept would be to include metabolomic data in the design and fitting of computational models. While there are many well-developed metabolic modeling strategies, most of these approaches have not yet been adapted to effectively leverage the additional information that metabolomics can offer. Nonetheless, these strategies have made substantial contributions to metabolic engineering. We discuss these computational approaches to establish how they have been used to date in metabolic engineering, to suggest how metabolomics can contribute to their effectiveness, and to highlight current efforts to integrate the two.

### 4.1. Constraint-Based Models

The most basic models for metabolic engineering use simplified equations for bioreactor kinetics to empirically fit relationships between characteristics such as metabolite uptake or secretion and specific growth rate. While these models are useful as tools for investigating specific behaviors of existing strains, their small-scale and coarse-grained nature precludes broader application to directing engineering strategies, as well as the possibility of substantively integrating metabolomics data even when available [13].

Early biochemical modeling strategies initially sought to move beyond such simplistic approaches by compiling knowledge of metabolic pathways and enzyme kinetics into detailed mechanistic models to predict the dynamic behavior of metabolite concentrations [119]. However, numerous issues hampered these efforts. Incomplete knowledge of the regulatory structure or the form of reaction rate equations can limit the accuracy of these types of models [120]. More importantly, the necessary kinetic parameters are for the most part unknown, or have only been measured *in vitro* for specific organisms (although recent efforts have sought to develop methods to determine kinetic parameters relevant to *in vivo* conditions via selected intracellular metabolite measurements [121,122]). Additionally, many models in systems biology also exhibit “sloppy” behaviors in regard to parametric sensitivity, where model performance is sensitive only to certain parameter combinations and consistent parameter estimation is difficult even with sufficient data [123].

To attempt to overcome these issues, “constraint-based” approaches that calculate metabolic fluxes primarily from stoichiometry were developed. This change of focus from dynamic metabolite levels to fluxes made sense, as the idea of optimizing and controlling metabolic fluxes has long been a fundamental part of metabolic engineering. These approaches allow flux calculations without the difficulties arising from parametric uncertainty by predicting flux distributions from the structure of the biochemical network and constraints on the feasible range of fluxes [124,125].

#### 4.1.1. Flux Balance Analysis: The Prototypical Constraint-based Model

Flux Balance Analysis (FBA) is, in short, a modeling technique that uses metabolic network stoichiometry, a set of feasible flux ranges, and a cellular objective function to calculate an optimal flux distribution for a metabolic network [124,125]. To achieve this, FBA invokes a pseudo-steady state assumption. (Based on the fact that metabolic reactions occur more quickly than upstream cellular processes like signal transduction, transcription, and translation, this may often be a reasonable simplification.) As a result, the non-linear set of differential equations describing the stoichiometric metabolite mass balance is reduced to a set of linear equations in terms of unknown reaction fluxes. Since the number of reactions exceeds the number of metabolites in biologically relevant metabolic networks, the system is underdetermined. Bounds on the solution space and an objective function are required to fully specify a unique solution. Selection of a linear objective function, such as maximization of a flux corresponding to biomass generation, specifies a linear programming (LP) problem (which can be efficiently solved numerically). By construction, FBA does not use information on metabolite concentrations themselves in its calculations. 

A few examples of basic FBA for metabolic engineering include estimation of flux distributions from MFA measurements [31,126], prediction of knockout performances to assess proposed strain designs [5], use of artificial (virtual) metabolites to better capture flux ratios from ^13^C MFA [127], systematic evaluation of different objective functions in *E. coli* [128], and evaluation of Elementary Flux Modes from flux measurements [129] and transcription datasets [118]. A study of *E. coli* metabolism comparing growth in aerobic and anaerobic culture by Chen *et al.* highlights the complementary nature of FBA and MFA [130]. They note that FBA often performs well when predicting extracellular fluxes, but intracellular flux distributions are more difficult; it is important to compare fluxes derived from FBA calculations against actual fluxes as derived from measurements and MFA. Tools such as the COnstraints Based Reconstruction and Analysis (COBRA) toolbox for MATLAB [131], CellNetAnalyzer [132], the Systems Biology Research Tool (SBRT) [133], and OptFlux [134] are available for simplified implementation of constraint-based modeling methods (and are listed in Table 1).

#### 4.1.2. Model Reconstructions

A prerequisite step in FBA is the reconstruction of genome-scale metabolic networks for the organism of interest. Reviews by Fiest *et al.* [135] and by Thiele and Palsson [136] describe this process in detail, and new strategies for efficiently or even automatically performing parts of this process are being continuously developed (listed in Table 1) [137,138,139,140]. The reconstruction process involves the synthesis of a genome-scale model from established biochemical and genomic knowledge, stored in publically accessible databases. These databases span various organisms and layers of biological information (e.g., biochemical pathways, transcription factors, and nutrient transport mechanisms). Examples of relevant databases for this process (as well as several for metabolomics in general) are also listed in Table 1.

A few recent examples particularly relevant to metabolic engineering and metabolomics include models of *A. niger* [141], *C. acetobutylicum* [138], and *Clostridium beijerinckii* [142], updated reconstructions of *E. coli* [143,144], and addition of lipid metabolism to a model for *S. cerevisiae* [145]. Notably, a reconstruction of *Mycoplasma genitalium* [126] has recently been incorporated into a whole-cell computational model by Karr *et al.* [146], who have suggested development of a similar model for *E. coli* as a possible next step. Network reconstructions for several dozen species are publicly available, and programs such as MetRxn have been developed to aid comparison across model reconstructions [147]. Some examples of these are shown in Table 1.

#### 4.1.3. Applications of Constraint-based Models in General to Metabolic Engineering

The original FBA framework has been supplemented with dozens of refinements broadly referred to as constraint-based models. While these models retain the optimization problem framework based on stoichiometric constraints, the flux constraints or objective function are altered. We direct the reader to reviews on the topic of FBA by Lee, Gianchanani, and others for more complete discussion of these methods [148,149], though it is instructive to analyze a few representative classes relevant to metabolic engineering. Furthermore, we note that these approaches do not generally make use of metabolite measurements.

The most basic refinements are straightforward extensions of FBA, from adding a simplified representation of transcriptional regulatory constraints, to integrating uptake/effluxes and comparing against extracellular concentration profiles. Examples from this family include regulatory FBA (rFBA) [150,151,152], dynamic FBA (dFBA) [153,154], integrated FBA (iFBA) [154,155], and integrated-dynamic FBA (idFBA) [156]. While these refinements demonstrate improved accuracy over basic FBA, most dynamic examples only make use of targeted extracellular concentration profiles as a means of constraining their dynamic elements. Using metabolomics data to constrain FBA solutions could provide these strategies direct information about intracellular metabolite levels in place of relying purely on the calculated fluxes to infer intracellular behaviors. Similarly, regulatory versions of FBA have looked at transcriptional regulation, but not regulation at the metabolic level (e.g., allosteric regulation). Metabolomics can provide information necessary to capture these effects.

Another class of refinements to FBA comprises methods intended to better reduce the discrepancy between model predictions and experimental observations. Optimal metabolic network identification (OMNI) is used to identify discrepancies between measured and predicted fluxes, and then determine changes that need to be made to the model to better match the measurements [157]. As a proof of concept, it identified bottleneck reactions that, once removed from the model, accounted for reduced growth rates in evolved *E. coli* strains compared to the original model predictions. The GrowMatch algorithm uses growth and lethality data to correct a network reconstruction [158], and its application to a reconstruction of *S. cerevisiae* led to improved knockout lethality predictions. [159]. A number of these corrections were only identified by taking into account double gene deletion data, and improvements included growth-medium specific regulatory constraints. These (and other [160,161]) methods would also directly benefit from the additional information that metabolomics datasets can provide about the intracellular state of metabolism.

More directly relevant to metabolic engineering applications is a class of refinements focused on predicting the result of metabolic network alterations. An early and well-known example of this is Minimization of Metabolic Adjustment (MOMA), which formulates a quadratic programming (QP) problem to find the feasible flux distribution nearest to the original FBA solution in response to a gene knockout [162]. OptKnock uses a bi-level optimization framework to balance an FBA objective function with a desired overproduction target [163] (the work by Hua *et al.* compares the results of an evolved strain against an OptKnock prediction [47]). OptGene uses a genetic algorithm to generate metabolic engineering strategies [164], and was used by Asadollahi *et al.* to design a strain exhibiting improved sesquiterpene production in *S. cerevisiae* [165]. Another extension is OptForce, which uses flux measurement data to generate a minimal set of engineering interventions required to guarantee the desired overproduction target [166]. In addition to recapitulating already proven strategies for succinate production in *E. coli*, it also identified several other successful and nonintuitive strategies. Again, while none of these methods integrate metabolomics data into their calculations, they could potentially be improved significantly if they could harness such data.

#### 4.1.4. Thermodynamic Constraints: Integrating Metabolomics Data into Constraint-based Models

As reviewed above, the majority of constraint-based modeling strategies make negligible use of systems-scale metabolite data in their calculations. The requirement that organisms adhere not only to stoichiometric mass conservation, but also to thermodynamic restrictions on energy and entropy, provides one means of introducing metabolite concentrations into the constraints. Several constraint-based model techniques make use of metabolite or metabolomics data in this fashion.

Network-embedded Thermodynamic (NET) Analysis combines pre-determined flux directions with quantitative metabolomics datasets and the metabolite Gibbs energy of formations to determine the feasible ranges of Gibbs free energy of reaction throughout the system [167]. This method can assess the internal consistency of a metabolomics dataset, predict thermodynamically feasible ranges for unmeasured metabolites, and identify putative sites of transcriptional regulation. anNET is a MATLAB implementation of the algorithm designed to facilitate straightforward application of NET analysis [168]. 

**Table 1:** Summary of Software Tools Presented in This Manuscript.

*Tool Name*	*Reference*	*Description*
**Metabolomics Data Processing**		
ChromA	[72]	GC-MS Peak Alignment
Metab	[75]	GC-MS Data Statistical Analysis Package
MetaboAnalyst 2.0	[78]	Web-based Metabolomics Data Processing Pipeline
MetAlign	[76]	GC-MS and LC-MS Data Processing Pipeline
Mzmine 2	[74]	MS Data Processing Pipeline
SpectConnect	[71]	GC-MS Peak Alignment
Xalign	[69]	LC-MS Data Pre-processing
XCMS Online	[77]	Web-based Untargeted Metabolomics Pipeline
**Constraint-Based Modeling **		
anNET	[168]	MATLAB-based NET analysis
CellNetAnalyzer	[132]	MATLAB-based Metabolic and Signal Network Analysis
COBRA Toolbox	[131]	MATLAB-based FBA Toolbox Suite
OptFlux	[134]	Open Source, Modular Constraint-based Model Strain Design Software Toolbox
Systems Biology Research Tool	[133]	Open Source, Modular Systems Biology Computational Tool
**Network Reconstruction **		
GapFind, GapFill	[137]	Automated Network Gap Identification and Hypothesis Generation
GeneForce	[139]	Regulatory Rule Correction for Integrated Metabolic and Regulatory Models
MetRxn	[147]	Web-based Knowledgebase Comparison Tool
Model SEED	[140]	Web-based Generation, Optimization and Analysis of Genome-scale Metabolic Models
**Databases**		
BioCyc	[169]	Genome and pathway database for >2000 organisms
BRENDA	[170]	Comprehensive enzyme database, ~5000 enzymes
ChEBI	[171]	Biologically relevant small molecules and their properties
KEGG	[172]	Genomes, enzymatic pathways, and biological chemicals
MetaCyc	[173]	>1,900 metabolic pathways from >2,200 different organisms
PubChem	[174]	Biological activity and structures of small molecules

NET analysis of a metabolomics dataset for *S. cerevisiae* revealed a thermodynamic inconsistency in whole-cell NAD/NADH ratio, which led Canelas *et al.* to engineer a strain with a bacterial mannitol-1-phosphate 5-dehydrogenase to allow them to determine cytosolic levels from measurements of cytosolic pH, fructose-6-phosphate, and mannitol-1-phosphate [175]. They found that the ratio calculated from this method was consistent in NET analysis and displayed a sensitive response to redox perturbations. Other studies have also used NET analysis to verify the thermodynamic consistency of their measurements in a variety of metabolic engineering contexts [9,59,176].

Henry *et al.* developed Thermodynamic Metabolic Flux Analysis (TMFA), a constraint-based modeling approach similar to NET analysis [177]. While both methods calculate the feasible Gibbs energy of reaction ranges in the network, TMFA does not require user-supplied flux directions and by design excludes thermodynamically infeasible futile cycles from the resulting calculations. (It is worth noting, though, that the elimination of infeasible futile loops from the FBA solution space can be performed separately and without concentration data [178,179]). Henry *et al.* also discuss the use of group contribution methods to calculate Gibbs energy of formation and the effect of the associated estimation uncertainties on TMFA results [177]. Recent improvements to group contribution methods should improve this approach’s accuracy [180,181].

Garg *et al.* applied TMFA to ^13^C MFA calculations to produce thermodynamically consistent fluxes [182]. The *E. coli* network reconstruction published by Fiest *et al.* includes thermodynamic information, and the manuscript includes an assessment of thermodynamic consistency using TMFA [143]. Canelas *et al.* used TMFA as one criterion for identifying near-equilibrium reactions in an effort to use metabolomics datasets to determine *in vivo* thermodynamic (and kinetic) parameters [54].

While TMFA and NET analysis have been the prevailing approaches used to incorporate metabolite concentrations and thermodynamic constraints into constraint-based modeling approaches, several other methods have been developed. For example, Bordel *et al.* developed a constraint-based model based on Ziegler’s principle for the maximization of entropy production that uses non-equilibrium thermodynamics to identify flux bottlenecks; they identified reactions that impeded previous efforts to improve glycolytic flux via overexpression of glycolysis enzymes in *S. cerevisiae* [183]. Hoppe *et al.* designed a constraint-based model that combines thermodynamic constraints similar to TMFA and NET analysis with a penalty function for deviations from concentration measurements. The minimization of both the overall flux and the penalty function are used as the optimization criteria. In applying this new approach, they found that increased network size leads to increased sensitivity of the resulting flux distribution to metabolite concentrations and Gibbs free energy changes [184]. 

### 4.2. Kinetic Models

Constraint-based models have successfully directed numerous metabolic engineering projects. However, by construction they often ignore or have trouble dealing with dynamic metabolite behaviors that may have significant impact on final product titers, and in general they only indirectly make use of metabolite concentration measurements. Improved knowledge of network structures and strategies for dealing with parametric uncertainty have made ordinary differential equation (ODE) based models of metabolic kinetics increasingly viable tools for strain design. These methods explicitly model intracellular concentrations, making them attractive and convenient frameworks for integrating metabolomics datasets. 

#### 4.2.1. Recent Developments in Kinetic Modeling Strategies

Kinetic models are built around explicit mathematical descriptions of enzyme-metabolite interactions. Natural choices for kinetic rate laws are mass-action kinetics and Michaelis-Menten kinetics, but a review by Heijnen highlights several approximate rate laws that require fewer parameters and are relevant to metabolic engineering applications [185]. Included are discussions of the S-system and power-law kinetic rate laws, long established by the early efforts at kinetic modeling that developed into Biochemical Systems Theory, and reviewed specifically in the context of metabolic networks recently by Voit [186,187,188,189].

In light of recent genome-scale reconstruction efforts, several investigators have sought to assess the properties of several of these approximate forms in the context of metabolic networks. Examples of this approach include study of the glycolytic pathway in *S. cerevisiae* using a local linearization method [190] and lin-log kinetics [191,192], as well as study of central carbon metabolism in *E. coli* to compare lin-log kinetics, convenience kinetics, power law kinetics, and Michaelis-Menten kinetics [193]. This last study found that mixed models of Michaelis-Menten and lin-log kinetics are well suited for large-scale networks where the true rate laws are often still unknown. While these studies are not examples of using kinetic models for metabolic engineering or with metabolomics *per se*, they demonstrate the principles underlying the effective implementation of kinetic modeling for those purposes.

#### 4.2.2. Examples of Kinetic Models for Metabolic Engineering

Independent of metabolomics, kinetic models have already been applied in several recent metabolic engineering contexts. Rasler *et al.* constructed a dynamical model of cellular redox state in *S. cerevisiae* to assess response to oxidative stress [194]. Literature kinetic values provided a qualitative fit to experimental data, and they achieved significantly improved quantitative matching by partially fitting parameter values (allowing them to vary by up to 4-fold from the literature values). Chassagnole *et al.* constructed a kinetic model of central carbon metabolism in *E. coli* in conjunction with MCA [120]. Their model captured most of the concentration trends, but even with parameter fitting, structural limitations of the model precluded complete agreement. A similar study by Oh *et al.* constructed a model of lactic acid fermentation in *Lactococcus lactis*, and the subsequent results were also used for MCA [20].

Ensemble approaches are also promising, and are in part a response to issues of parametric “sloppiness” which can preclude precise determination of kinetic parameters [123]. These entail constructing a set of models that are structurally identical, but each using a different parameter set. Each model fits the training data comparably well, and the behavior of the whole ensemble is used to make predictions. Tran *et al.* developed a model for central metabolism in *E. coli* as a proof of concept [195]. Starting with an ensemble spanning the entire thermodynamically feasible range of parameter combinations, they observed convergence to consistent fold-change behavior as training data were introduced. This effort led to an ensemble model constructed by Rizk *et al.* that predicted the effect of gene knockouts on the production of aromatic compounds in *E. coli* [196], and a model by Contador *et al.* to predict flux data in L-lysine-producing *E. coli* [197]. While these examples do not use metabolomics, we note that metabolomics datasets could in principle be easily adapted to this method and would be quite well-suited for further constraining the parameter ensembles.

#### 4.2.3. Integrating Metabolomics Datasets into Kinetic Models

None of the aforementioned ODE models explicitly look to integrate metabolomics data into their analyses, due in part to the only recent development of more quantitative metabolomics techniques. While scarce, there have been some efforts to use metabolomics data to improve or exploit ODE-based models of metabolism. For example, Klimacek *et al.* used published kinetic parameters with time-course metabolomics measurements to assess metabolic control in xylose-fermenting *S. cerevisiae* [9]. Similarly, a model of nitrogen assimilation in *E. coli* developed by Yuan *et al.* used kinetic parameters from the literature together with a genetic algorithm to fit undefined parameters to metabolomics data [60]. Proteomic and transcriptomic data were also used to adjust reaction rate predictions. While these examples both use metabolomics data, the modeling strategies focused only on capturing the dynamics of individual pathways and modules – not the whole metabolic network.

Two recent examples have attempted to apply metabolomics measurements to models of an entire metabolic network. Yizhak *et al.* developed a constraint-based modeling approach they referred to as integrative “omics”-metabolic analysis (IOMA), which solves a QP problem in a constraint-based model to fit a flux distribution to proteomic and metabolomics data [198]. Their approach introduces a reaction rate model based on Michaelis-Menten kinetics, and uses proteomic data in conjunction with metabolomics data to fit the kinetic parameters and satisfy the steady-state flux requirement in the unperturbed system. This results in a defined system of ODEs. When compared against FBA and MOMA to predict the effects of gene knockouts based on an erythrocyte kinetic model and published data for *E. coli*, IOMA demonstrated significantly improved recall, precision, and accuracy.

A mass action stoichiometric simulation (MASS) modeling strategy described by Jamshidi *et al.* follows a different scheme, by fitting thermodynamic equilibrium constants to a measured metabolomics dataset and a calculated flux distribution [199]. The kinetic rate law equations are then solved to obtain the forward reaction rate constants. As a proof of concept, they used this method to construct a human erythrocyte model, and compared it against a previously published model. They also included mechanisms to account for regulation of five enzymes using mass-action kinetics. They assessed metabolite and protein level trajectories in response to redox load simulations, and performed sensitivity analysis to determine the dominant time scales within the model. The MASS model demonstrated many of the key behaviors of the original erythrocyte model.

Notably, these last two methods both take advantage of constraint-based modeling strategies, but result in ODE-based kinetic models that can subsequently be used for strain design. This reflects the complementary nature of metabolite fluxes and concentrations, especially when faced with system-wide parametric uncertainty. However, to fully capture the wide-ranging dynamics that directly and indirectly contribute to the often subtle and nonintuitive behaviors exhibited in engineered strains, additional model detail is necessary. More advanced modeling strategies will need to find ways to integrate additional information, including proteomics and transcriptomics, to meet this need.

## 5. Conclusions

Metabolomics is the global analysis of the metabolic content of a living system. While it has found increasing application in fundamental biological research and in fields of clinical interest (e.g. disease biomarker discovery), there is surprisingly little use of metabolomics approaches to drive metabolic engineering efforts. Existing experimental approaches to supplement rational metabolic engineering efforts typically focus instead on the determination of flux with MFA techniques, or the use of enzyme assays and analytical platforms such as HPLC for highly targeted metabolite measurements. 

While global analysis methods have been used to better predict and assess the effects of metabolic engineering modifications, the techniques most typically used have been transcriptomic or proteomic analyses – not metabolomics. While this may have previously been due to the relative immaturity of metabolomics techniques, the current technology in the field should allow for easy integration of metabolomics into metabolic engineering workflows.

Direct applications of metabolomics datasets to metabolic engineering include expanding the existing narrowly targeted analysis methods to a broader scope, identifying non-intuitive mutations in strains produced by directed evolution, and adding direct metabolic context to other global analysis datasets. Computational approaches have also begun to integrate metabolomics datasets through thermodynamic constraints in constraint-based models, or even more directly in the case of some kinetic models.

However, long-term strategies will need to find novel ways of incorporating the system-wide perspective provided by metabolomics and other global analysis methods. Such approaches will facilitate strain design based on increasingly detailed mechanistic descriptions and enable us to engineer strains towards any arbitrary product, not just those well-suited to high-throughput screens and directed evolution. Computational methods have a great deal of potential here. In the case of kinetic models, combining the metabolome and proteome can help address issues of *in vivo* parameter estimation. Ensemble models are proving to be one effective method of addressing issues of parametric uncertainty and model “sloppiness”, and metabolomics provides substantial data to better constrain feasible parameter sets. With proper alterations and structural changes, constraint-based models may be able to more explicitly incorporate metabolite concentrations into constraints to capture effects such as allosteric regulation. We expect that these and other recently introduced approaches to integrate metabolomics data will yield substantial improvements in the efficiency and accuracy of prospective strain designs, both accelerating the pace and expanding the scope of developments in metabolic engineering. Nonetheless, it is clear that the depth and breadth of metabolic insight afforded by metabolomics is currently not being sufficiently exploited, and it is likely in such advances that we will make our most significant next steps as metabolic engineers.
